# Implementation of co-designed strategies enhances referral to pulmonary rehabilitation for people with COPD in tertiary care: an interrupted time series study

**DOI:** 10.1186/s13012-026-01512-4

**Published:** 2026-06-10

**Authors:** Sarah Hug, Vinicius Cavalheri, Daniel F. Gucciardi, Richard Norman, Christine Teague, Natasha Bear, Kylie Hill, OPTIMAL consortium, Nola Cecins, Tenae Cowie, Tamara Hatton, Jade Larsson, Caitlin Vicary, Carol Watson, Peta Winship

**Affiliations:** 1 Curtin School of Allied Health, Faculty of Health Sciences, Curtin University, GPO Box U1987, Perth, 6845 Australia; 2 Department of Physiotherapy, Royal Perth Hospital, Perth, Western Australia Australia; 3 Allied Health, South Metropolitan Health Service, Perth, Western Australia Australia; 4 Exercise Medicine Research Institute, Edith Cowan University, Perth, Western Australia Australia; 5 Curtin School of Population Health, Faculty of Health Sciences, Curtin University, Perth, Australia; 6 Institute for Health Research, Notre Dame University, Perth, Western Australia Australia

**Keywords:** Co-design, Referral, Pulmonary rehabilitation, COPD

## Abstract

**Background:**

Pulmonary rehabilitation programs (PRPs) are a core component of care for people with chronic obstructive pulmonary disease (COPD), yet referral is low. The aims of this study were to; (i) engage with people with COPD receiving care at a tertiary hospital and HCPs involved in the management of people with COPD, to co-design and implement strategies that sought to improve referrals to PRPs; (ii) evaluate the effect of implementing co-designed strategies on the rate of referral to PRPs across three tertiary hospitals; and (iii) understand the sustainability of co-designed strategies by estimating relative costs and number of potentially prevented hospitalisations to achieve cost neutrality.

**Methods:**

This uncontrolled quasi-experimental study was informed by the behaviour change wheel and co-design framework. It included three distinct periods: (i) pre-implementation, (ii) co-design and implementation, and (iii) post-implementation. During pre- and post-implementation, people with COPD were sequentially recruited from three tertiary hospitals. Medical records were tracked to determine PRP referral. During the co-design and implementation period, four people with COPD and six HCPs developed and implemented strategies addressing known barriers to PRP referral. Referral rates in pre- and post-implementation periods were compared using segmented Poisson regression. Sustainability was estimated by modelling the number of hospitalisations that would need to be avoided annually to offset implementation costs. Program uptake, completion, and clinical outcomes were not assessed.

**Results:**

Co-designed strategies included PRP promotional products; off-loading parking expenses; deploying ‘case finders’ across the three tertiary hospitals whose primary role was to find people with COPD appropriate for a PRP and support them to engage; and visual prompts for HCPs to refer. Data were available on 590 people with COPD (age 69 ± 11 years, FEV_1_ 49 ± 20% predicted, 56% males). Between pre- and post-implementation periods, the mean referral rate to PRP increased from 25% to 52%, respectively (incident rate ratio 2.1, 95% confidence interval 1.1 to 3.9). Co-designed strategies would be cost neutral if they prevented 14 major or 30 minor COPD-related hospitalisations annually.

**Conclusions:**

Co-designed strategies that addressed known barriers to people with COPD being referred to a PRP produced a two-fold increase in rate of referrals. Given the evidence that PRPs reduce healthcare utilisation, long-term funding of these strategies may indicate cost neutrality.

**Supplementary information:**

The online version contains supplementary material available at 10.1186/s13012-026-01512-4.

## Contributions to the literature

• Demonstrates how co-design and behavioural frameworks can be applied to improve referral practices to pulmonary rehabilitation from tertiary care.•Provides real-world evidence that theory-informed, person-centred strategies can double referrals to evidence-based pulmonary rehabilitation.•Highlights the value of engaging both patients and healthcare professionals in service improvement initiatives.•Underscores the need for ongoing partnering with consumers and monitoring of referral, uptake, and completion rates to sustain quality improvement.•Suggests future directions for enhancing communication skills to address psychological barriers and support patient engagement

## Background

Chronic obstructive pulmonary disease (COPD) is a common, costly condition characterised by persistent breathlessness that limits participation in activities of daily living [1]. In 2023, COPD was estimated to cost the Australian community AUD$832 million, accounting for 18% of the national respiratory disease expenditure [2], with similar trends of high burden throughout North America, the United Kingdom and Europe [3]. Treatment aims to reduce symptoms, improve exercise tolerance and health-related quality of life, and reduce healthcare utilisation [1]. There is robust evidence such benefits can be achieved with pulmonary rehabilitation programs (PRPs) [4–6]. Despite PRPs being endorsed by international respiratory societies as standard care for people with COPD [1], referral, uptake and completion of these programs is low [7].

For people with COPD to achieve the benefits of pulmonary rehabilitation, they must first be referred by a healthcare professional (HCP). In Australia, programs have eligibility criteria such as having objective evidence of a pulmonary disease and being symptom-limited during physical activity [8]. Prior to initiating a referral, HCPs require prospective candidates to express some willingness to accept this referral [8]. A scoping review of 28 observational studies involving 7,271 participants with COPD and 760 HCPs involved in the management of people with COPD across primary and tertiary care revealed a median referral rate of 17% [9]. Barriers to referral are commonly reported to include lack of awareness of the program and referral pathways, transport difficulties, and the perception of the person being too sick to exercise [9, 10]. Earlier work has demonstrated that amongst people who were broadly appropriate for a referral to a PRP, the only factor that separated those who were referred from those who were not was the person’s interest in the program [11]. In-depth qualitative analyses of interviews with people with COPD regarding their perception of barriers to accepting a referral demonstrated marginally explored psychological barriers (such as feeling unworthy of healthcare, an inability to make sense of how exercise training might reduce dyspnoea, and long-standing activity avoidance) were potent barriers reducing people’s willingness to accept a referral to a PRP [12]. This work questions the current understanding that geographical challenges, such as parking and transport difficulties are the most salient barriers to attending a PRP. Indeed, if this was true, we would expect home or telehealth models, which do not incur parking or travel-related problems, to have optimal referral rates and nearly perfect uptake. Whilst referral rates to home or telehealth models of PRP have not been reported, the uptake was no better than centre-based programs (62% for home-based and 68% for centre-based) [7, 13].

Given the overwhelming evidence supporting PRPs, especially in people with COPD [4], international respiratory societies have called for action towards research that improves the utilisation of these programs [14], of which, supporting referral is the important first step.

## Methods

### Research aims

The aims of this study were to:

1. Engage with people with COPD receiving care at a tertiary hospital and HCPs involved in the management of people with COPD, to co-design and implement strategies that sought to improve referrals to PRPs;
2. Evaluate the effect of implementing co-designed strategies on the rate of referral to a PRP across three tertiary hospitals;
3. Understand the sustainability of co-designed strategies by estimating relative costs and number of potentially prevented hospitalisations to achieve cost neutrality.

### Study design and setting

This was a two-year quasi-experimental study, informed by the behaviour change wheel [15] and conducted across three tertiary hospitals in Perth, Western Australia. It comprised three distinct periods; (i) pre-implementation, (ii) co-design and implementation, and (iii) post-implementation [16]. Approval was obtained from relevant Human Research Ethics Committees.

### Sampling and study procedures

#### Pre- and post-implementation period (people with COPD)

Recruitment and data collection for the pre- and post-implementation period occurred between August 2020 and January 2021 and March and August 2023, respectively. Participants were sequentially recruited during a hospital admission and from respiratory outpatients. To be eligible to participate, people needed to be broadly appropriate for a referral to a PRP. These criteria, consistent with usual clinical practice in Australia [8], comprised: spirometric evidence of COPD, being limited by symptoms of dyspnoea or fatigue during activities of daily living, ability to exercise without physical assistance, ability to consistently follow instructions, having a life expectancy of greater than 6-months, and not residing in supported residential care [16]. Once recruited, referrals to a PRP were prospectively tracked using hospital-based electronic referral systems, medical records, and via discussions with the participant or the HCP responsible for offering a PRP. For participants recruited during an admission, referrals were prospectively tracked for 2-weeks after hospital discharge and, where applicable, 2-weeks after their post-discharge outpatient appointment. For participants recruited during an outpatient appointment, referrals were prospectively tracked for 2-weeks following this appointment. Data were recorded in the Research Electronic Data Capture system [17]. Participants recruited during the pre-implementation period were not followed into the post-implementation period; new participants were recruited during the post-implementation period.

#### Co-design and implementation period (people with COPD and HCPs)

Recruitment and data collection for the co-design and implementation period occurred between July and November 2021. Aligning with established co-design methodology (Additional file 1, Table S1) [18], we sought to recruit participants to the co-design period who were representative of the end-user populations (people with COPD and HCPs who manage people with COPD in tertiary care). Participants with COPD were purposively recruited from the pre-implementation cohort. We sought to recruit people with differing characteristics (e.g., disease severity, age, gender and socioeconomic background) and referral experiences (e.g., referred to a PRP and attended, not referred, or declined a referral). Healthcare professionals who researchers interacted with during the pre-implementation period were eligible if they referred people to a PRP. We sought to recruit various disciplines involved in the management of people with COPD (e.g., respiratory and general medicine physicians, respiratory nurses and physiotherapists, HCPs who provide a PRP, and community health providers). Participants with COPD and HCPs who expressed interest and willingness to actively participate in the co-design component (including being able to attend and actively contribute to co-design workshops and complete assigned homework tasks) were recruited to the co-design team.

Five iterative workshops were facilitated by SH using an established co-design methodology [18]. Each workshop ran for 2 hours at a local community hall identified as accessible to participants. The content of each workshop was iterative, reflexive and messy (Table 1). Initial workshops were opportunities for the group to get to know each other. Personal experiences and a summary of the findings of previous studies [11, 12, 19] were shared, with objectives set. In subsequent workshops, the facilitator encouraged reflection and activities to meet objectives. Co-design and implementation occurred concurrently. As strategies to address barriers were developed in the early workshops, they were implemented in real time across the three hospitals, with all strategies in place before the end of the five month period. A steering committee, comprising HCPs from each site, supported implementation and ensured that strategies were delivered as intended across the three tertiary hospitals. Participants with COPD were remunerated for their involvement during this period.

**Table 1:** Summary of workshops that occurred during the co-design and implementation period

Workshop	Date	Number of attendees	Overview of workshop content and activities	Homework
1	24 July 2021	10 + scribe	Obtained written informed consent **Aim = Getting to know you and our purpose** Introductions – Why are you here and what do you want out of this process? A sense of belonging – terms of reference created that outlined ground rules, expectations and responsibilities Facilitator shared the intent of the workshops; the purpose and objectives were defined **Activity:** Build capacity of co-creators by sharing the primary findings from – reflections were discussed Co-designers shared their own experiences with referrals to a PRP – discussion **Activity: **Knee-jerk ideas for solutions to the problem presented were brainstormed on butchers’ paper	Reflect on today’s information – What can we do about it? What has been tried in the past? Ask your family/friends/colleagues. Document your thoughts and share with the group via email or bring to the next session − 5-minute summary (max) Everyone to consider: Think about a utopian world where funding isn’t a barrier; what can we do to help people with COPD get referred pulmonary rehabilitation? Brainstorm and bring along next time Come up with a group name Consider who else you might like to invite to these meetings Facilitator to circulate minutes and summarise the terms of reference
2	21 August 2021	10 + scribe	Workshop 1 reflection: terms of reference confirmed **Aim = What will our strategies be? How will they look?** **Activity: **Broke into groups of 3 and brainstormed solutions. Considered utopian society (if you had free rein to improve referrals and uptake, what would you do?) **Activity**: Data-prompted scenarios: Photo of person hospitalised with an exacerbation COPD: “Would you expect this person to think about exercise during their admission?” Photo of person walking to their outpatient appointment: “This person is on their way to an outpatient appointment at a tertiary hospital. How can they advocate for a referral to pulmonary rehabilitation?” Provided an example of a clinic letter: “What information do you get from this letter? How can it be improved? What would entice people to come to their appointment?” (reflect on your own experience) Shared low-hanging fruit ideas from earlier studies – reflection on their utility	Design your own pamphlet/poster – consider the content Brainstorm what the volunteer role description needs to be Have a look over summary of brainstormed solutions – what do you like, which can we use, which do we need to change? Have a quick look at short videos and decide if they are to use or not. Come armed with catchy slogans for posters, pamphlets, t-shirts, etc. Design a logo Consider what information we need to deliver in education programs for HCPs
3	4 September 2021	7 + scribe	Workshop 2 reflection **Aim = (i) Decide which strategies we are pursuing, and (ii) What do you want them to look like?** Confirmed which interventions we are pursuing **Activity: **As a group, designed and developed the interventions: - PRP pamphlet - Letter to your colleague with COPD - Poster - Video – script/storyboard - HCP t-shirts - Doctor’s computer prompts - Stamp/sticker for inpatient medical records - Feedback about referral behaviours - Volunteer advocate job description **Activity: **advocate role brainstorming (what is it? who can do it? what are their duties? etc.) Increasing visibility of PRP letters on health software program Education (physiotherapy, medical, nursing) Developing a standard practice for decliners Catchy phrases for t-shirts/adverts/banners Uptake of referral Send pamphlet with appointment letter Volunteer to phone person who has been referred and ensure they actually come Physiotherapist to phone patient within 2 weeks of referral **Activity:** defined where these interventions are to be used	Take intervention prototypes home to family/friends and trial them → report back on what worked well and what we can change Dot point script/storyboard for video Facilitator to approach multimedia departments of the three tertiary hospitals for support with branding of the posters Facilitator to organise production of logo Facilitator to source filmmaker for video Facilitator to present summary of interventions to steering committee for feedback
4	2 October 2021	7 + scribe	Workshop 3 reflection **Aim = Finalise interventions, specifically:** **(i) volunteer role** **(ii) posters** **(iii) storyboard and script for video + source people to be involved** **(iv) testimonial** **(v) infographic/pamphlets** Poster and pamphlet prototypes from multimedia presented and discussed Video script brainstormed and finalised Discussed where the interventions should be placed Co-designers confirmed they were happy for implementation to commence	For facilitator: liase with steering committee to implement each of the strategies across the three tertiary hospitals
5*	27 November 2021	4 + scribe	Workshop 4 reflection **Aim = Reflect on the process** Finalised interventions presented to co-design team Feedback from steering committee provided – unable to implement medical record stamps (due to medicolegal issues) or volunteers (due to governance issues). Back up: case finder HCPs **Activity:** Define the case finders’ approach and role **Activity: **Reflection. Discussion – Have we met our objectives? What were your motivations to do this? What worked well? What needed to change? Do you feel like you made a difference? How? What difference has this made to you? Co-designers shared reflections of the process **Discussion: **Where to from here? Co-designer team congratulated for their commitment and work over the past five months Gratitude expressed by the facilitator for everyone’s engagement	Facilitator to work with steering committee to implement co-designed interventions across Perth’s tertiary care

Footnotes: Number of attendees includes the facilitator (SH). *Final workshop was delayed due to longer than anticipated time to implement interventions after workshop 4

Abbreviations

COPD – chronic obstructive pulmonary disease

HCP – healthcare professional

PRP – pulmonary rehabilitation program

The determinants of behaviour and active ingredients that each co-designed strategy targeted were mapped to the behaviour change wheel [15]. The acceptability, practicality, effectiveness, affordability, spill-over effects, equity (APEASE) criteria [15] were applied to understand the utility of each strategy. The cost of delivering each strategy was recorded.

### Statistical analyses

Data were analysed as an interrupted time series (ITS) [20] with the primary outcome being rate of referral to a PRP. To assess changes in the rate of referral between pre- and post-implementation periods, data were aggregated into fortnightly intervals. This resulted in 13 pre-implementation and 12 post-implementation intervals. The discrepancy in the number of intervals was due to the varying length of calendar months where recruitment occurred. The rate of referral for each interval was calculated as the number of people with COPD who were referred to a PRP within 2-weeks of recruitment, expressed as a proportion of the number of people with COPD recruited to the study. Segmented Poisson regression was used to compare rate of referrals across intervals, evaluate the level change between pre- and post-implementation, and predict trends [20, 21]. Methodological issues such as autocorrelation, seasonality and overdispersion were assessed [20].

**Fig. 1:**
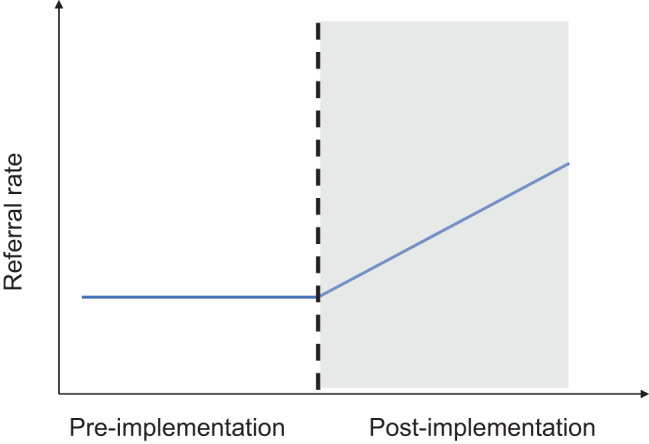
Proposed impact model for the interrupted time series with segmented Poisson regression analysis

As the ITS is an exploratory analysis [20], formal sample size estimation was not required [22]. Experts recommend that researchers pre-specify the expected direction and nature of change [20]. We hypothesised that the implementation of co-designed strategies would lead to a gradual increase in referrals to pulmonary rehabilitation during the post-implementation period (Fig. 1). The results are presented as an incident rate ratio (IRR) and 95% confidence interval (CI). All data were analysed using Stata 16.1 (StataCorp, College Station, Texas).

To understand the sustainability of co-designed interventions, we conducted a cost–benefit analysis. First, we estimated the annual cost of implementing the co-designed strategies (A), and the pre-implementation annual cost of delivering a PRP in Western Australia, per person, per class (B). Then, considering (B) and using the change in rate of referrals between the pre- and post-implementation periods, we estimated the cost per additional person referred to PRP (C). Then the cost of co-designed strategies (A) and the cost per additional person referred (C) were compared to the 2025 Australian national cost of a COPD-related hospitalisation [23]. From this, we estimated the annual number of hospitalisations that would need to be prevented for co-designed strategies and additional PRP delivery to be cost neutral (D). Further information regarding net cost impact analysis is provided in Additional file 2. 

## Results

**Fig. 2:**
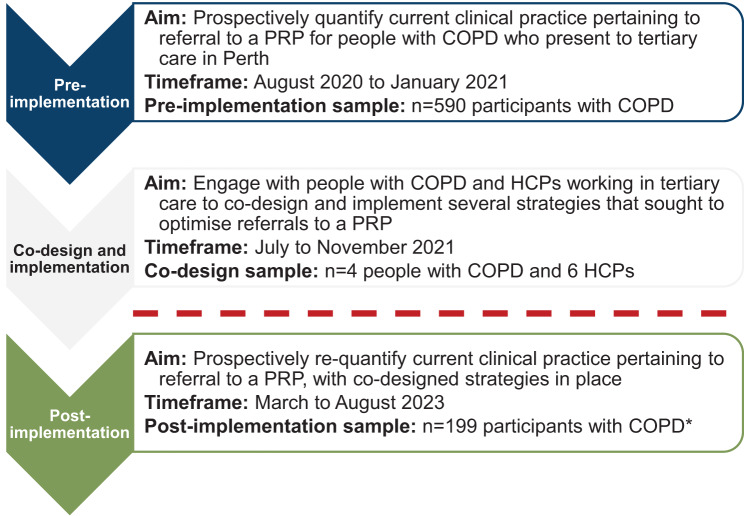
Timeline and recruitment of three study periods. Footnotes: *new participants were recruited during post-implementation. Red dotted line indicates period that the study paused due to community transmission of COVID-19 in Western Australia

A timeline of study periods and recruitment samples are provided in Fig. 2.

### Pre-implementation period

During the pre-implementation period, 682 people with COPD were approached. Of these, 412 consented and were eligible, and 391 were analysed. Reasons for exclusion from analysis were participants passing away (*n* = 17), withdrawing (*n* = 3) or being lost to follow-up (*n* = 1). During the pre-implementation period, 97 (25%) participants were referred to a PRP within their respective 2-week window.

### Co-design and implementation period

**Fig. 3:**
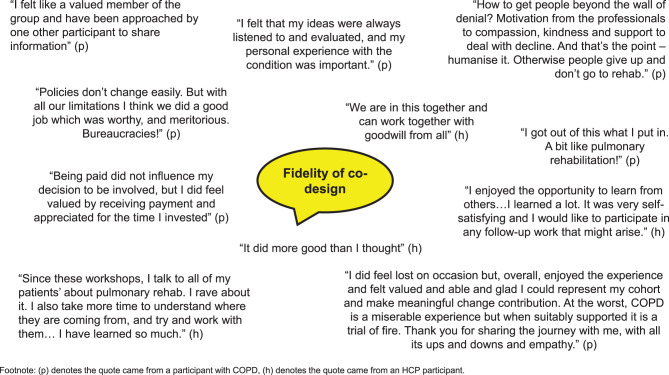
Demonstrating fidelity of the co-design process

Four people with COPD, six tertiary care HCPs involved in providing care to people with COPD and one researcher were recruited to the co-design team (Table 2) and invited to attend five workshops (Table 1). Attendance across workshops varied between stakeholder groups. All four participants with COPD attended all five workshops, whereas healthcare professionals attended fewer sessions (Table 2). This reflected both the iterative nature of the co-design process—where some HCPs were invited later to provide specific expertise—and the impact of competing clinical demands that limited HCPs ability to attend all workshops. The co-designed strategies targeted at changing referral acceptance of people with COPD are presented in Table 3, whilst those targeted at changing referral practices of HCPs are presented in Table 4. Strategies targeted at changing behaviour of both of these groups are presented in Table 5. For each strategy, these tables describe: (i) the most prominent barriers or enablers to referral addressed [24]; (ii) relevant intervention function and/or policy category [15]; (iii) active ingredients [25, 26]; (iv) the mode of delivery [27]; and (v) sites implemented (or reasons for non-implementation). Logistics of implementation for each strategy are provided in Additional file 1 (Table S2). To understand their utility, each implemented strategy were mapped to the APEASE criteria (Table 6) [15]. Fidelity of the co-design process was authentically captured through observation and reflection (Fig. 3). No adverse events were observed with the implementation of co-designed strategies.

**Table 2:** Demographics of the co-design team

Representative of	Gender	Background	Referral information	Prior involvement	Number of workshops attended
Person with COPD	Male	Retired engineer and lecturer	Declined a referral during an admission for an exacerbation of COPD. Attended PRP after recruitment	Pre-implementation period and qualitative study participant	5
Person with COPD	Female	Part time bookkeeper	Referred during an admission and current PRP participant	Pre-implementation period and qualitative study participant	5
Person with COPD	Female	Retired academic	Referred during an admission and current PRP participant	Pre-implementation period and qualitative study participant	5
Person with COPD	Female	Retired librarian	Advocated for a referral during outpatient appointment, current PRP participant	Pre-implementation period and qualitative study participant	5
Healthcare professional	Female	PRP physiotherapist at a secondary hospital	Receives referrals, delivers hospital-based PRP	Correspondence during the pre-implementation period	4
Healthcare professional	Female	PRP and cardiac rehabilitation physiotherapist at a tertiary care hospital	Receives referrals, delivers hospital-based PRP	Correspondence during the pre-implementation period, qualitative study participant	4
Healthcare professional	Female	Respiratory advanced trainee	Referrer	Qualitative study participant	2
Healthcare professional	Male	Respiratory consultant	Referrer	Correspondence during the pre-implementation period	2
Healthcare professional	Female	Community-based physiotherapist	Referrer, delivers home-based PRP	Correspondence during the pre-implementation period, qualitative study participant	2
Healthcare professional	Female	Respiratory nurse (community care)	Referrer	Qualitative study participant	1
Researcher	Female	PhD candidate, acute care physiotherapist at a tertiary hospital	Referrer	Conducting the research	5

Abbreviations

COPD – chronic obstructive pulmonary disease

PRP – pulmonary rehabilitation program

**Table 3:** Summary of co-designed strategies targeting people with COPD mapped to the behaviour change wheel

Strategy or process change	Target barrier (b) or enabler (e) [21]	Intervention function or policy category [14]	Active ingredients (BCT) [22, 23]	Mode of delivery [24]	Sites implemented
‘Breathing buddies’ (volunteer patient PRP advocates)	Social influences of others with COPD who have completed a PRP (e) Subjective norms of what is acceptable (b) Attitudes towards exercise or group exercise (b) Motivation to commence exercise in the context of dyspnoea (b)	Education, persuasion, modelling and enablement	3.1 Social support (unspecified) 6.1 Demonstration of the behaviour 6.2 Social comparison 9.1 Credible source 10.8 Incentive (outcome) 15.3 Focus on past success	Provider: person with COPD who has graduated from a PRP and/or is participating in a maintenance program Format: in person or over the phone Setting: present in inpatient respiratory wards and outpatient respiratory clinics to talk to prospective PRP candidates; have a mobile number that prospective candidates can call if they don’t want to interface in person Tailoring: convey a personalised story to prospective PRP candidates (a lay, lived-experience perspective to encourage participation), answer questions, offer support	Nil – unable to achieve consensus among steering committee of how to operationalise this (who has oversight of the volunteers, particularly beyond study completion) and limited by study timeframes
Case finders	Attitudes towards exercise or group exercise (b) Beliefs about capabilities and consequences of exercise (b) Social influences of HCPs (e) Perceived susceptibility/ vulnerability associated with exercise in the context of dyspnoea (b) Self-image and physical capabilities (b)	Education, persuasion and enablement	These varied and were individualised to the patient’s needs. Examples included: 3.1–3.3 Social support (unspecified, practical and emotional) 5.2 Salience of consequences 6.3 Information about others’ approval 7.7 Exposure 8.7 Graded tasks 9.1 Credible source 9.3 Comparative imaging of future outcomes 10.6 Non-specific incentive 13.2 Framing/reframing 13.3 Incompatible beliefs 15.1 Verbal persuasion about capability 15.3 Focus on past success 16.3 Vicarious consequences	Provider: HCPs Format: in person or over the phone. Subtle differences in how case finders generated referrals to a PRP across the three hospitals are provided in Additional file 1 Setting: inpatient and outpatient settings one to two days per week; primary role = to find people with COPD, initiate a discussion about a PRP, and ensure they are referred to the program Materials: case finder toolkit (Additional file 1) Tailoring: personalised approach with purposeful focus on building the therapeutic alliance. Approached with empathy, curiosity and unconditional regard; use lay language; engage in a two-way conversation about a PRP and invest time to explore and unpack internalised perceptions impeding engagement; follow up where necessary to offer a PRP again	All
Infographic (describing a PRP and testimonials of PRP graduates)	Knowledge of PRPs and what they entail (b) Attitudes towards exercise and group exercise (b) Social influences of others with COPD (e) Perceived susceptibility/ vulnerability associated with exercise in the context of dyspnoea (b)	Education and persuasion	4.1. Instruction on how to perform behaviour 5.1. Information about health consequences 6.3 Information about others’ approval 9.1 Credible source 13.2 Framing/reframing	Provider: HCPs (referrers, case finders, PRP physiotherapists) Format: A5 infographic Setting: available in clinic waiting rooms; mailed out to patients along with preprogram assessment appointment letter; provided during a discussion about a PRP Tailoring: can be used during interactions with prospective PRP candidates to share vicarious experiences of past PRP candidates	All
InReach PRP liaison	Social influences of HCPs (e)	Persuasion, enablement and modelling	3.2 Social support (practical) 3.3 Social support (emotional) 9.1 Credible source	Provider: tertiary PRP physiotherapists (with experience of program delivery) Format: in person and where required, followup over the phone Setting: inpatient respiratory wards Tailoring: find patients who are admitted with an exacerbation and provide information about the program and support referral acceptance (see tailoring for case finders)	Nil – no capacity from PRP teams/service delivery to meet this demand (staffing scarce due to COVID19)
Modify the information provided on appointment letters	Environmental context and resources (b)	Environment restructuring, service provision	4.1 Instruction on how to perform the behaviour 5.1 Information about health consequences	Amend the appointment letters to remove technical aspects (i.e. the clinic code). Instead provide a lay and clear description of the purpose of the appointment	Nil – unable to change the information as letters are automatically generated from hospital referral booking system
Other resources made available: – LFA fact sheets – Living better with exercise booklet	Knowledge of PRPs and what they entail (b) Environmental context and resources (b)	Education and enablement	4.1. Instruction on how to perform behaviour 5.1. Information about health consequences 8.1 Behavioural practice/rehearsal	Materials: in addition to co-designed resources, visibility and use of resources already available to HCPs was also increased Setting: available in clinic waiting rooms and on respiratory wards for physiotherapists to provide to people with COPD	All
Sharing patient testimonials of a PRP	Social support and social influences of others with COPD who have completed a PRP that increase perceived credibility, emotional engagement, and normative pressure (e.g., “People like me changed, so I can too”) (e)	Persuasion	9.1 Credible source 3.1 Social support (unspecified) 3.2 Social support (practical)	Providers: HCPs (referrers, case finders, PRP physiotherapists) Format: handwritten ‘letter to your comrade with COPD’ Setting: available in clinic waiting rooms; mailed out to patients along with preprogram assessment appointment letter; during a discussion about a PRP Tailoring: can be used during interactions with prospective PRP candidates to offer vicarious experiences	Nil – Australian health practitioners regulation agency cautioned against the use of testimonials
Video	Social influences of others with COPD (e) Beliefs about consequences of exercise (e) Perceived susceptibility/ vulnerability associated with exercise in the context of dyspnoea (b) Intentions to commence exercising to better one’s health (b)	Education, persuasion and modelling	5.1 Information about health consequences 6.1 Demonstration of the behaviour 6.2. Social comparison 10.8 Incentive (outcome) 13.2 Framing/reframing	Provider: referring HCPs Format: video on display in clinic waiting room and inpatient televisions. Case finders had an iPad with the video loaded Setting: outpatient clinic waiting room; inpatient wards; during a case finder discussion about a PRP Tailoring: case finders can show it to prospective PRP candidates via iPad to demonstrate a patient’s journey through the program	Was delivered by case finders in waiting rooms (i.e. not on hospital TVs)
Opt-out referrals from respiratory inpatients [Process change]	Memory, attention and decision processes of HCPs (b)	Service provision and enablement	12.1 Restructuring the physical environment 7.1 Prompts/cues	All patients admitted to a respiratory ward with an exacerbation of COPD are to be automatically referred to a PRP on discharge. Exceptions are if residing in HLCNH, cognitively impaired or unable to ambulate independently prior to admission	Nil – unable to achieve consensus among steering committee of how to operationalise this
Flexible programs [Process change]		Regimented days and times of programs (b)	Service provision	12.1 Restructuring the physical environment	Provider: PRP service within tertiary care Materials: after hours employment of physiotherapists to enable scheduling of classes Tailoring: offer face-to-face programs that are delivered after hours and on weekends to enable people who work full time to access them around work hours
Provide PRP information within 2-weeks of referral [Process change]	People forget they have been referred to the service (b) Appointment letter does not provide information about the service (b)	Service provision	5.1 Information about health consequences 7.1 Prompts/cues	Mail out appointment letter and infographic within two-weeks of referral receipt	All
Standard practice for follow-up of people who decline a PRP referral [Process change]	Stages of change – motivation can change over time (b and e)	Service provision	1.2 Problem solving 3.1–3.3 Social support	Case finder to follow up via phone call at an agreed upon time to discuss barriers to and options for engagement	All
2-week phone call for those referred [Process change]	People forget they have been referred to the service (b) Appointment letter does not provide information about the service (b) Lack of opportunity to ask questions about what is involved (b)	Service provision	1.1 Goal setting 3.1–3.3 Social support 5.1 Information about health consequences 7.1 Prompts/cues	Provider: PRP physiotherapists Format: over the phone Setting: outpatients Tailoring: make phone contact with patients referred prior to their initial appointment to introduce self, reiterate the program, notify them of the appointment, answer questions, and encourage attendance	All

Footnotes: ^*^Availability of staff after hours is scarce (both to deliver a program, and to escalate if issues occur during a class)

Abbreviations

BCT – behaviour change technique

COPD – chronic obstructive pulmonary disease

HCP – healthcare professional

HLCNH – high level care nursing home

LFA – Lung Foundation Australia

MoA – mechanism of action

PRP – pulmonary rehabilitation program

**Table 4:** Summary of co-designed strategies targeting healthcare professionals, mapped to the behaviour change wheel

Strategy or process change	Target barrier (b) or enabler (e) [21]	Intervention function or policy category [14]	Active ingredients (BCT) [22, 23]	Mode of delivery [24]	Sites implemented
Education to rotating staff	Knowledge of what PRP entails, eligibility criteria and how to refer (b) Skills pertaining to referral practices (b)	Education, modelling, training and enablement	4.1 Instruction on how to perform behaviour 4.2 Information about antecedents 6.1 Demonstration of the behaviour 9.1 Credible source	Provider: PRP physiotherapists Format: in person Setting: tertiary care professional development session Tailoring: education delivered to rotating medical staff, newly rotated ward physiotherapists and respiratory nurses about the program, eligibility criteria and how to refer; share a patient experience through the program, and encourage referrers to attend an assessment and/or PRP class of a patient they have referred to follow their journey, interact with current class candidates to hear the benefits first-hand, observe what is involved	All – Educational resources updated and education rolled out, however, HCPs attending an assessment or class was not completed
Feedback about referral behaviours via live dashboard	Memory, attention and decision processes (b) Behaviour cueing to enter a discussion about a PRP referral (e) Feedback processes about referral practices (e) Motivation to refer patients to a PRP (e)	Enablement and environmental structuring	2.2 Feedback on behaviour 7.1 Prompts/cues 12.3 Restructuring the physical environment	Provider: PRP physiotherapists Format: electronic dashboard circulated via weekly emails/on ward TVs Setting: tertiary hospital Tailoring: place a dashboard in respiratory clinical areas of weekly referral numbers generated from each of the three tertiary hospitals	Nil – unable to create a live dashboard within study timeframes, also no clarity of how to operationalise this beyond the study
PRP letters available on digital medical record program [Process change]	Behaviour cueing to enter a discussion about a referral (b)	Environmental restructuring	7.1 Prompts/cues 12.5 Adding objects to the environment 12.3 Restructuring the physical environment	Increase the visibility of PRPs by allowing letters to be visible on the electronic viewing system	All
Referral nudges	Memory, attention and decision processes (b) Behaviour cueing to enter a discussion about a PRP referral (e)	Environmental restructuring	7.1 Prompts/cues 12.5 Adding objects to the environment	Setting: outpatient clinics Format: electronic system-automated prompts for referral Tailoring: For inpatients – have a popup when someone with COPD is admitted with a respiratory complaint to discuss pulmonary rehabilitation For outpatients – have a popup prompting the HCP to discuss a PRP during the consult	Nil – inability of electronic system to meet this request. This was amended to physical prompts detailing eligibility criteria and how to refer to a PRP, which were placed by clinic and ward computers that HCPs use
Stamp/sticker for inpatient notes and outpatients	Memory, attention and decision processes (b) Behaviour cueing to enter a discussion about a referral (e)	Environmental restructuring	1.2 Problem solving 1.4 Action planning 7.1 Prompts/cues 12.5 Adding objects to the environment	Provider: ward/clinic clerks Format: stamp/sticker to be placed in the medical record of a patient with COPD Setting: inpatients and outpatients Materials: stamp to include the following documentation: - diagnosis - breathless on physical activity (Yes/No) - suitable (Yes/No) - referred (Yes/No) - reason - if declined, method for following up Tailoring: use as a prompt to engage in a discussion about a PRP with a patient with COPD	Nil – unable to achieve consensus among steering committee of how to operationalise this, and medicolegal concerns raised

Abbreviations

BCT – behaviour change technique

COPD – chronic obstructive pulmonary disease

HCP – healthcare professional

PRP – pulmonary rehabilitation program

**Table 5:** Summary of co-designed strategies that target *both* people with COPD and healthcare professionals, mapped to the behaviour change wheel

Strategy or process change	Target barrier (b) or enabler (e) [21]	Intervention function or policy category [14]	Active ingredients (BCT) [22, 23]	Mode of delivery [24]	Sites implemented
Poster (prompting a discussion about a PRP)	Environmental context – PRPs are not visible on wards in clinics (e) Memory, attention and decision processes – HCPs forget to consider PRPs for their patients with COPD (b)	Environmental restructuring and education	4.1 Instruction on how to perform behaviour 5.1 Information about health consequences 7.1 Prompts/cues 12.5 Adding objects to the environment	Setting: on the wall in respiratory clinic waiting rooms Format: large poster Tailoring: designed to encourage people with COPD to ask their treating HCP about a PRP	All
Promotional t-shirts	Environmental context – PRPs are not visible on wards in clinics (b) Memory, attention and decision processes – HCPs forget to consider PRPs for their patients with COPD (b)	Environmental restructuring, modelling and persuasion	3.1 Social support, unspecified 6.1 Demonstration of the behaviour 6.3 Information about others’ approval 7.1 Prompts/cues 12.5 Adding objects to the environment	Provider: worn by HCPs who offer referrals to a PRP Setting: inpatients and outpatients Tailoring: used as a visual aid to encourage discussions about PRPs not just with patients, but also among referrers (e.g. discuss eligibility criteria, increase service visibility, increase connections between referrers and the PRP service)	All Note: primarily worn by physiotherapists
Change the name ‘pulmonary rehabilitation’ [Process change]	Uncertainty about the program as its label does not give an indication of what is involved (b)	Service provision	13.2 Framing/reframing 12.2 Restructuring the social environment	Rebrand the name of the program to something understandable for people with COPD	Nil – awareness raised but too difficult to explore and operationalise during study timeframes

Abbreviations

BCT – behaviour change technique

COPD – chronic obstructive pulmonary disease

HCP – healthcare professionals

PRP – pulmonary rehabilitation program

n/a – not applicable

**Table 6:** Co-designed interventions, mapped to the APEASE criteria (presented alphabetically) [15]

Intervention	Acceptability	Practicability	Effectiveness	Affordability	Spill-over effects	Equity
Case finders	High	Medium	High	Yes	Positive	Increase
Education to rotating staff	High	High	High	Yes	Positive	Increase
Infographic (describing a PRP and testimonials of PRP graduates)	High	High	High	Yes	None	Increase
Poster (prompting a discussion about a PRP)	High	High	High	Yes	None	Increase
Promotional t-shirts	High	High	High	Yes	Positive	Increase
Provide PRP information within two-weeks of referral	High	Medium	High	Yes	Positive	Increase
PRP letters available on digital medical record program	High	High	High	Yes	Positive	None
Referral nudges	High	High	High	Yes	None	None
Standard practice for follow-up of people who decline a PRP referral	Medium	Low	High	Yes	Positive	Increase
Two-week phone call for those referred	High	Medium	High	Yes	Positive	Increase
Video	High	High	High	Yes	Positive	Increase

Footnotes: The APEASE criteria definitions and grading are as follows

Acceptability – Is this intervention function acceptable to important stakeholders, e.g. the target population, those delivering the intervention, funders? High/medium/low

Practicality – Is this intervention function able to be targeted at the required scale, with the required quality for as long as will be required? High/medium/low

Effectiveness – Will targeting this barrier/facilitator achieve the policy objectives and provide value for money?High/medium/low

Affordability – Can using this intervention function be achieved within an available budget? Yes/no

Spill-over effects – What effects, good or bad, will using this intervention function have beyond the target behaviour?Positive/negative/none

Equity – What impact will using this intervention function have on health and social inequities? Increase/decrease/none

Abbreviations: PRP – pulmonary rehabilitation program

### Post-implementation period

During the post-implementation period, 472 people with COPD were approached. Of these, 210 consented and were eligible, and 199 were analysed. Reasons for exclusion from analysis were participants passing away (*n* = 7) or withdrawing (*n* = 4). During the post-implementation period, 103 (52%) participants were referred to a PRP within their respective 2-week window. A “case finder” was a trained individual tasked with identifying people with COPD who were suitable for pulmonary rehabilitation in tertiary care. Their co-designed role included engaging in conversations about pulmonary rehabilitation, identifying and addressing individual barriers to referral and uptake, and advocating for referrals (Additional file 1, Table S2). Of those participants referred during the post-implementation period, 94% interacted with a case finder for a median of 15 minutes (interquartile range 10 to 25 minutes).

### Segmented poisson regression analysis

**Fig. 4:**
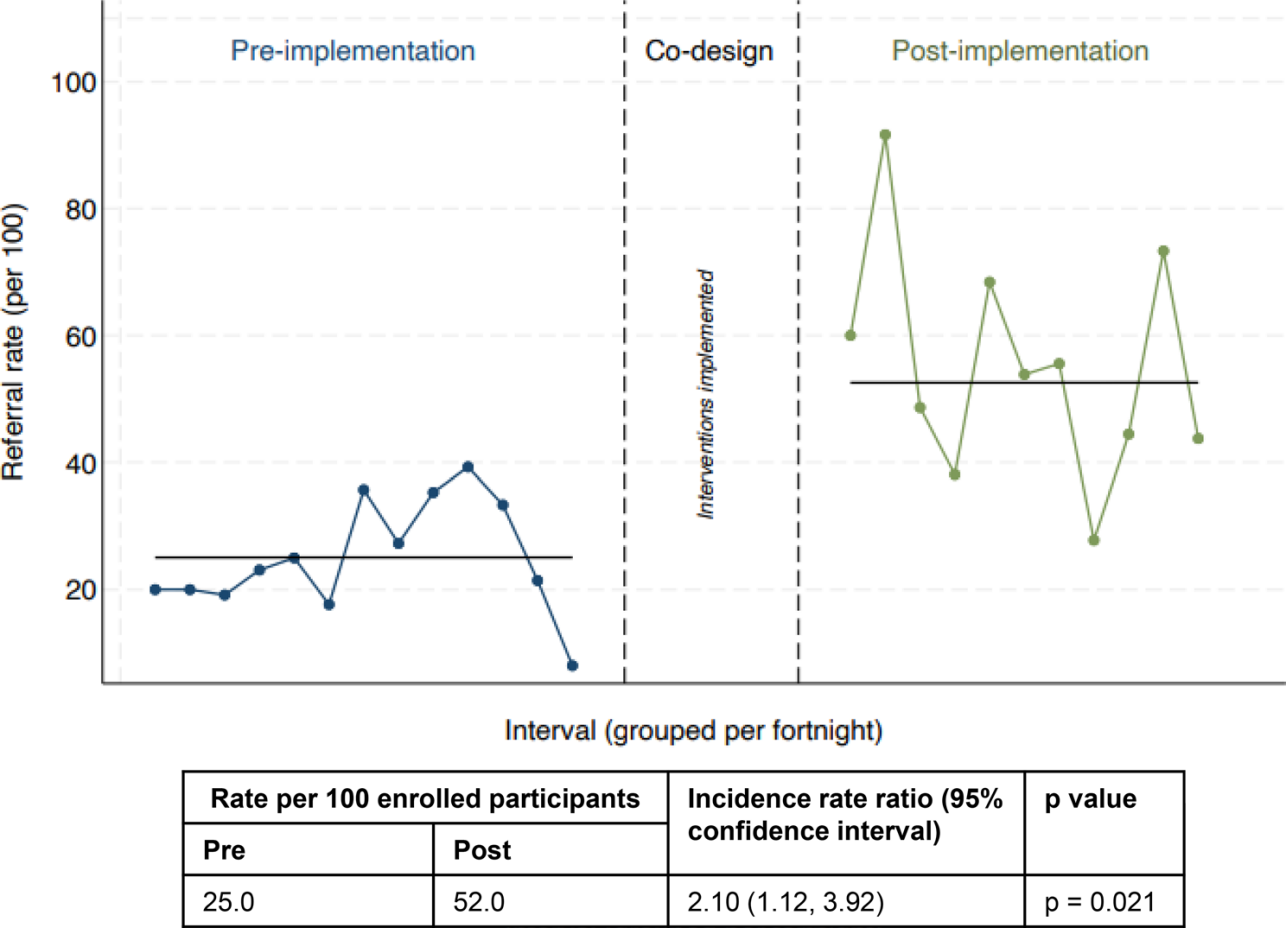
Interrupted time series with segmented Poisson regression of rate of referrals to a PRP between the pre- and post-implementation periods

Considering both pre- and post-implementation recruitment periods, a total of 1,154 people with COPD were approached. Of these, 622 consented and were eligible, and data were available on 590 (mean ± standard deviation or n [%]; age 69 ± 11 years, forced expiratory volume in one second [FEV_1_] 49 ± 20%, body mass index 28 ± 8 kg/m^2^, 328 [56%] males, 356 [60%] recruited as an outpatient). The final Poisson regression analysis evaluated a change in level of referrals from pre- to post-implementation. An interaction term between time and period was not significant (*p* = 0.34) and was excluded from the regression equation. Compared with the pre-implementation period, the post-implementation period was characterised by a two-fold increase in the rate of referrals to a PRP (IRR 2.1, 95% CI 1.1 to 3.9; Fig. 4).

### Net cost impact

The annualised cost of implementing the co-designed strategies was AUD$147,975 (A). The estimated annual cost of delivering a PRP during the pre-implementation period was AUD$20,092, with costs per person, per class (B) ranging between AUD$20.09 to AUD$28.70 (Additional file 2).

We estimated that across a 5-month period, there would be 295 people with COPD broadly eligible for a PRP who present to Perth’s tertiary care for management of their COPD (average number recruited over the pre- and post-implementation periods – i.e. 391 + 199/2). This equates to 59 people per month and a total of 708 eligible people who present to tertiary care per year. Applying the pre- and post-implementation referral rates to this annual estimate yields 177 referrals per annum at the rate of referral measured during the pre-implementation period (708 × 0.25), and 368 referrals per annum at the rate of referral measured during the post-implementation (708 × 0.52). This represents an increase of 191 referrals per year attributable to co-designed strategies (Additional file 2).

Considering (B), the estimated cost of providing a PRP to these additional 191 people referred (C) is between AUD$3,837 (191 × AUD$20.09) and AUD$5,485 (191 × AUD$28.70) per annum, depending on attendance. Considering (A) and (C), we estimated that an allocation between AUD$151,812 to AUD$153,460 per annum would be needed to cover both the cost of the co-design strategies and PRP delivery for the 191 additional people. Factoring the cost of COPD-related hospitalisations in Western Australia [23], to achieve cost neutrality (D), it would be necessary to prevent either 14 major or 30 minor COPD-related hospitalisations per year (Additional file 2).

## Discussion

This was a pragmatic, quasi-experimental study conducted in the real-world tertiary care setting. It actively engaged people with COPD and HCPs to optimise referrals to evidence-based PRPs in adults with a complex chronic disease. To our knowledge, this study is the first to achieve a doubling in the proportion of people with COPD who appeared broadly appropriate for a PRP who were actually referred to a PRP from tertiary care.

This study focused intentionally on referral behaviour, as this represents the greatest point of attrition in the pulmonary rehabilitation pathway [9, 11, 28] and was the behavioural target identified during co-design. Behaviour change theory [15] emphasises the importance of addressing individual behaviours discretely, and our co-designed strategies were developed specifically to influence the referral decision rather than subsequent steps such as uptake, adherence or completion. While these downstream outcomes are important, they require distinct behavioural targets, stakeholders and co-design processes, and were therefore beyond the scope of the present study.

Key strengths include sequentially recruiting and prospectively tracking participants to determine if they had been referred to a PRP. The ITS study design is a robust method to understand longitudinal change [20, 29] because it reduces the chance that intervention effects being confounded by secular trends quantified during the pre-implementation period. We acknowledge that without a control group who were denied access to these co-designed strategies, we cannot exclude the possibility of other external factors occurring around the time of the intervention, influencing the observed increase in referrals. However, given geographical constraints of tertiary healthcare in Western Australia, other trial designs (e.g., cluster randomised controlled trial) were impractical. Another strength was the use of iterative co-design workshops that were guided by theoretical frameworks [15, 18] to develop strategies to improve referrals that were authentic and meaningful to both people with COPD and the HCP who provide care for them within Perth’s context. While some strategies may not be generalisable as they were developed to address behavioural determinants and system constraints specific to Perth’s tertiary care context, the co-design process offers a model for improving PRP referrals in other settings.

The main limitations of this study pertained to recruitment. Specifically, there was difficulty in recruiting people with COPD to the pre- and post-implementation periods. Of those approached (*n* = 1,154), 622 (54%) consented to participate and of these, data were available on 590 (95%). Although suboptimal, a recruitment rate of 54% is unusually high in this clinical population, who are notoriously difficult to engage. In randomised controlled trials that recruit people with COPD, the recruitment fraction rarely exceeds 25% [30]. The 54% consent rate reflects the broader challenge of engaging people with COPD in research, with individuals from marginalised or less health literate backgrounds more likely to decline participation, thereby constraining representativeness. Furthermore, despite efforts to recruit a diverse co-design group, people from marginalised backgrounds, culturally diverse groups, or those with lower health literacy were underrepresented, reflecting the broader challenge that those least engaged in healthcare are also the hardest to engage in research. This may have influenced the types of strategies developed during co-design, including the emphasis on promotional materials that may appeal more to motivated, health literate individuals. While the co-design group acknowledged this limitation and sought to develop culturally safe and accessible interventions, the limited diversity of participants remains a constraint on the generalisability of strategies developed. Additionally, the focus on people with COPD in this study may also mean these results are not generalisable to people with other chronic respiratory diseases. Although recruitment occurred during the COVID-19 pandemic, community transmission was negligible in Western Australia due to strict, government-imposed border closures. The only implication of the pandemic was that restrictions imposed when borders re-opened precluded us from matching recruitment periods. Referrals to a PRP should be instant (i.e., generated at point of hospital discharge or during a clinic review). The two-week window for a referral was selected to allow for delays in referral initiation and ensured consistent followup of a referral across participants. We acknowledge this may have underestimated referrals made outside this period.

The improvement in the rate of referral to a PRP (25% to 52%) is considerably larger than any previously reported effect on referral. Specifically, a systematic review of 13 studies that explored generic strategies (e.g. delivering education to HCPs, improving referral procedures, a COPD discharge bundle that included PRP referral, patient-held scorecard to support discussions, and a co-designed PRP video) to improve the rate of referrals to PRPs from primary and tertiary care have reported improvements ranging between 6 and 15% [28]. Although we did not seek to identify which co-designed intervention was most influential, it is notable that during the post-implementation period, most people who were referred to a PRP had interacted with a case finder. These case finders were HCPs who were willing to undertake this role. There was no requirement to be a physiotherapist, or to have experience in the delivery of a PRP. All case finders were provided with standardised training to adopt a personalised approach to engaging with prospective PRP candidates, informed by earlier work (Additional file 1: (Table S2) [12, 19]. Specifically, qualitative work has demonstrated that willingness to accept a referral to a PRP was negatively influenced by deeply held unhelpful beliefs related to uncertainty, shame and feeling unworthy of healthcare [12]. When coupled with a HCP who lacks internalised value, interest and basic understanding of a PRP, it is very unlikely that someone with COPD will agree to accept a referral [12]. Case finders were coached to invest time in building a therapeutic alliance by understanding the individuals experience, their unhelpful beliefs, and offering tailored information about the PRP. It is possible that this personalised approach, with intentional focus on how we interact with people to build a therapeutic relationship, contributed to the higher referral acceptance rate observed in the study. The value of a strong therapeutic alliance has been identified as pivotal in the successful management of other clinical populations characterised by chronic noxious sensations such as low back pain [31]. Future research could consider targeting psychological beliefs held by people with COPD—as part of a treatable trait’s paradigm—to support engagement with health-enhancing behaviours such as a PRP.

Implementing the co-designed strategies posed several challenges. Some healthcare professionals expressed resistance for them, concerned that successful outcomes would increase their workload. Additionally, bureaucratic hurdles prevented the adoption of several novel strategies [32]. The inability to implement all strategies undermined the egalitarianism of consumer engagement, resulting in wasted time and resources and potentially limiting generalisability.

Finally, people with COPD known to a tertiary hospital who require specialist care are at the greatest risk of hospitalisation due to an exacerbation. Across Australia in 2017–18 there were 77,754 COPD-related hospitalisations, of which, 6,499 occurred in Western Australia [33]. This suggests that even small improvements in referral and subsequent uptake and completion of a PRP has the potential to influence health system costs. Our exploratory cost analysis was intended to illustrate these potential implications rather than provide a definitive estimate of cost effectiveness. As we did not collect data on PRP uptake, adherence or completion, we cannot assume that increased referral directly translates into reduced exacerbations or hospitalisations. The analysis therefore relies on established evidence that completing PR reduces hospitalisation risk [5, 34, 35], and should be interpreted cautiously. A more comprehensive economic evaluation would require detailed data on program completion and clinical outcomes, but the present findings highlight the potential for future work to examine these broader system level impacts.

## Conclusions

This pragmatic, quasi-experimental study conducted in the real-world tertiary care setting actively engaged with people with COPD and HCPs to optimise referrals to PRPs for many adults with complex chronic disease. Several co-designed strategies were implemented to enhance referral acceptance among people with COPD and improve HCP referral practices. These strategies yielded a two-fold increase in PRP referrals. While exploratory cost analysis suggests that preventing COPD-related hospitalisations could offset the cost of implementing these strategies, this should be interpreted cautiously, as the analysis relies on assumptions about downstream behaviours (e.g., PRP uptake and completion) that were beyond the scope of this study. Overall, this study offers a robust, person-centred approach to enhancing referral practices to a PRP. Ongoing efforts are needed to monitor and improve referral practices and optimise other aspects of the PRP journey.

## Electronic supplementary material

Below are the links to the electronic supplementary material.

Supplementary Material 1

Supplementary Material 2

Supplementary Material 3

## Data Availability

The datasets generated and analysed during the current study are not publicly available due restrictions from the Human Research Ethics Committee. Requests to view these data can be made to the corresponding author and ethics committee on reasonable request.

## Abbreviations

COPD: Chronic obstructive pulmonary disease
CI: Confidence interval
HCP: Healthcare professional
ITS: Interrupted time series
PRP: Pulmonary rehabilitation program

## Declarations

### Members of the OPTIMAL consortium

Nola Cecins^7^, Tenae Cowie^8^, Tamara Hatton^7^, Jade Larsson^2^, Caitlin Vicary^7^, Carol Watson^2^ and Peta Winship^8^

^7^Department of Physiotherapy, Sir Charles Gairdner Hospital, Perth,Western Australia, Australia

^8^Department of Physiotherapy, Fiona Stanley Hospital, Perth, Western Australia, Australia
