# Supplementary material for: Implementation of co-designed strategies enhances referral to pulmonary rehabilitation for people with COPD in tertiary care: an interrupted time series study
Source: Implement Sci. 2026 Jun 10;21:46. doi: 10.1186/s13012-026-01512-4 (PMC13483743; doi:10.1186/s13012-026-01512-4)
Supplement: Supplementary file 1 [file 13012_2026_1512_MOESM1_ESM.docx]

**Supplement 1 – Co-design and implementation information**

**Table S1.** Application of the PRODUCES framework (co-design methodology) prior to commencing co-design.^1^

| Problem  What was the reason for the process? | Target behaviour: Successful referrals to a PRP from tertiary care in Perth  Target population: People with COPD who are known to tertiary care, and HCPs who deliver tertiary COPD care  There is robust evidence to demonstrate that PRPs are important for people with COPD, but referral to and uptake of these programs is suboptimal, representing a failure of research translation into clinical practice. Earlier work that sought to optimise referral to and uptake of these programs has only achieved modest effects. ^2, 3^  Specific to the context of Perth, WA, the implementation gap is quantified in a prospective observational study. ^4^ Perspectives of barriers to PRP referrals are reported in an in-depth qualitative study. ^5^ |
| --- | --- |
| Objective  What was the aim of the process? | The intent for this process of co-design was to empower collaborations among people with COPD (consumers) and HCPs (end-users) to co-create field test and implement strategies that optimise referrals to a PRP for people with COPD known to tertiary care in Perth, thereby enabling more people to benefit from this highly evidence-based intervention. |
| Design  What specific methodology was used for co‑creation? | The BCW was used to guide the construction of knowledge in this study. The BCW encourages quantitative and qualitative methods, but it does not include participatory methods. Therefore, to allow for consumer and HCP engagement, the BCW was adapted so that stakeholders could engage with co-design. Based on the WA mental health engagement framework, the research team aimed for this process of co-design to sit across the ‘doing with’ domain of engagement. ^6^ |
| End-Users  Who will use the co‑created intervention? | Inpatients:   (i) People with COPD admitted with an exacerbation to respiratory or acute medical wards at one of the three tertiary hospitals in Perth, who appear broadly suitable for a PRP (ii) HCPs working on respiratory and acute medical wards at one of the three tertiary hospitals in Perth, and involved in the management of people with COPD; e.g. physiotherapists, doctors (consultants, advanced trainees, registrars, registered medical officers), nurses, other allied health professionals   Outpatients:   (i) People with COPD attending a respiratory medicine outpatient appointment at one of the three tertiary hospitals in Perth for management of their COPD, who appear broadly suitable for a PRP (ii) HCPs working in respiratory outpatients’ clinics at one of the three tertiary hospitals in Perth, and involved in the management of people with COPD; e.g. respiratory physicians (consultants, advanced trainees, registrars, registered medical officers), nurses and physiotherapists in clinic (as available) |
| Co-creators  Who engaged in the process?  Define the “actors,” their description and their expertise. | People with COPD and HCPs were recruited to the co-design team. S.H served as a facilitator of the co-design team, as well as an intermediary between the co-design team and the steering committee. Intermediation was required: (i) to include perspectives of the steering committee regarding possible interventions; and (ii) so members of the steering committee could support the implementation of co-designed interventions into clinical practice at the three tertiary hospitals.  The co-design team comprised four people with COPD, six HCPs involved in managing people with COPD in Perth and the facilitator. Co-creators provided insights to their lived experience and needs with relation to referral to a PRP.  The steering committee included HCPs and academic researchers (n = 24) who were not involved in the co-design workshops, but contributed ideas for interventions, and supported implementation of co-designed interventions into clinical practice. |
| Evaluation  How was success measured? | Success was measured through: (i) an informal evaluation of the process (fidelity); and (ii) the interrupted time series analysis to understand the effect of co-designed interventions. |
| Scalability  How can the developed solution(s) be scaled to a population level? | A distributed model for scaling was anticipated, ^1^ whereby interventions were fit for purpose and therefore clustered at a local level. Regarding scalability to a wider population (i.e. beyond Perth), as these interventions were designed at a local level^7^ and were highly situated for our PRP jurisdiction, the generalisability and applicability to external jurisdictions is unclear. Given the intricacies of individual health systems, the success of this study demonstrates that future endeavours to optimise referral to PRPs, and other behaviours of the PRP journey, need to be co-developed with people with COPD and HCPs at a local level.  Where feasible, co-designed interventions were left in clinical practice at the conclusion of the study, and made freely available on the open science project page for this study^8^ |

Abbreviations:

BCW – behaviour change wheel

COPD – chronic obstructive pulmonary disease

HCP – healthcare professional

PRODUCES – PRoblem, Objective, Design, (end-)Users, Co-creators, Evaluation and Scalability

PRP – pulmonary rehabilitation program

WA – Western Australia

**Table S2.** Co-designed strategies that were implemented across the three tertiary hospitals (presented alphabetically, and including logistics of implementation)

| Strategy | Implementation information | | | |
| --- | --- | --- | --- | --- |
| Case finders  *Targeting people with COPD* | The aim was to have case finders at each site one or two days per week (to align with ‘busy’ clinics where most patients with COPD are managed). A brief job description is provided below. Case finders received standardised training from researchers (KH and SH) to undertake this role. This training was based on our clinical experience and the findings of our previous quantitative and qualitative studies investigating referral behaviours in Perth’s tertiary hospitals,^9,4,5^ and placed emphasis on understanding a person’s perspective. That is, we encouraged a personalised approach that centred around understanding the person first and forming a therapeutic partnership that enabled case finders to safely explore the person’s specific barriers to engaging in a PRP. We encouraged case finders to be purposeful and invest time to actively listen, engage with and relate to the people they interact with.^10,11^ As some case finders had no experience with a PRP, we provided opportunities to build knowledge, skills and confidence in understanding what these programs involve. We provided standardised education to case finders around how pulmonary rehabilitation can benefit people with COPD. Case finders also experienced its benefits firsthand. That is, they got to know the local PRP physiotherapists and were provided with opportunities to watch an initial assessment and exercise classes and interact with current PRP candidates to understand their experiences. In addition to supporting case finders to internalise the value for PRPs,^11^ we also offered suggestions for ways to communicate with patients and tailor the information about these programs to support behaviour change.^11,12^ Suggestions aligned with motivational behaviour change techniques^12^ and included approaching with empathy, curiosity and unconditional regard; use of lay language; engage in a two-way conversation about a PRP and taking time to explore and unpack internalised perceptions hindering engagement. However, this was left largely to the case finders’ discretion to navigate, based on how the individual presented. Among people who declined a referral, case finders were able to follow up where necessary to offer a PRP again. Case finders were also provided with a toolkit that comprised co-designed materials as well as already available resources that they could use, at their discretion, to support patient engagement.^8^ There were subtle differences in the role of these case finders, with regards to PRP referrals across each of the three hospitals:  Site A: Case finders could email the tertiary PRP physiotherapist with the information about the prospective PRP candidate (in the form of a referral). PRP physiotherapists would generate an eReferral to their own service. Where case finders were clinicians that worked at this site, they could generate eReferrals to the relevant PRP service themselves.  Site B: When a case finder interacted with a prospective PRP candidate who was appropriate to be referred (but had not already been referred), the case finder needed to prompt the treating HCP to generate an eReferral.* Therefore, in the inpatient setting, case finders needed to prompt the ward physiotherapist. In the outpatient setting, case finders needed to prompt the treating physician before the end of clinic.  Site C: Case finders could email the tertiary PRP physiotherapist with the information about the prospective PRP candidate (in the form of a referral). PRP physiotherapists would generate an eReferral to their own service.  **Case finder job description**   - To ‘find’ cases of COPD who are suitable for pulmonary rehabilitation across respiratory outpatient clinics, and inpatient respiratory +/– general medicine wards - Provide information and education about the PRP service (this includes providing a ‘pack’ – pamphlet, infographic, contact information of service referred to) - Approach with compassion and empathy, no judgement around stigma associated with COPD, have the time to listen and answer patient questions. - Identify individual patient barriers to referral/uptake of pulmonary rehabilitation and collaborate with the patient to overcome these barriers. For example, provide parking/taxi vouchers, organise a buddy to attend with the patient, engage psychologist, etc.   - Identify and address unhelpful beliefs the patient has towards their diagnosis, exercise and physical activity, e.g. fear avoidance, catastrophising, protective behaviours and/or shame/stigma. – engage psychologist where necessary to support engagement with the pulmonary rehabilitation service - Gather relevant information (spirometry results, six-minute walk test results, etc.) and advocate clinicians to complete an eReferral to a PRP OR facilitate an eReferral yourself - Ensure the co-designed strategies are in place across the sites – replenish stocks when low, tweak as needed. Use the resources as part of your role - Link with current/past PRP participants who are willing to share their experience through the program to assist people to attend - Wear the promotional t-shirt while on site - Follow up patients flagged by ward/clinic staff, i.e. people who have declined, and try to get them to engage with the program as per above - Liaise with pulmonary rehabilitation physiotherapists:   - Follow up those who do not attend their initial appointments/poor class attendance/decliners – identify and assist patients to overcome their individual barriers so that they can attend/complete the program   - If has not been already done, contact those who have been referred to the service to check in; see if they have any questions about the service and encourage initial appointment attendance - Further roles as you see fit to achieve the aim of improving referral and uptake of pulmonary rehabilitation among people with COPD from the inpatient and outpatient respiratory settings - Keep a record of patients reviewed, intervention/s provided, outcome - Keep a record of your interactions and outcomes with patients/clinicians. Information can include:   - Date/time   - Who flagged this patient for review by the case finder?   - Inpatient / outpatient?   - Has this patient declined a referral to a pulmonary rehabilitation program during this recruitment period?   - What did you do?   - Is your input related to improving referral or uptake or both?   - What was the outcome? e.g. patient contacted and discussed the program – referral accepted, facilitated uptake, referred to rehabilitation in the home instead of hospital- or community-based program.   - Any follow-up required and by who?   - How has the case finder positively influenced referral/uptake for this patient?   - Do you have any other reflections? e.g. what’s working well, what’s not working.   - How many people you interact with and time you spend interacting with each patient | | | |
| Promotional t-shirts  *Targeting both people with COPD and HCPs* | Worn by HCPs who refer people with COPD to a PRP from tertiary care, and respiratory medicine and PRP physiotherapists.    | | | |
| Infographic  *Targeting people with COPD* | Infographics were placed in respiratory outpatient clinic waiting rooms, respiratory offices, and were provided to HCPs working in respiratory medicine and case finders to provide to prospective PRP candidates as they deemed appropriate. Infographics were also sent out with each PRP initial appointment letter.   | | | |
| Posters  *Targeting both people with COPD and HCPs* |  |  |  | |
|  | Posters were placed on the wall in respiratory outpatient clinics and on respiratory wards at each site. | | | |
| Video  *Targeting people with COPD* | Video playing on outpatient clinic television (<https://youtu.be/8dsZikSwVpg>). Where televisions were not able to display the video, the above poster was shown instead. Using an iPad, case finders showed the video to prospective PRP candidates during their interactions, where they deemed clinically appropriate. | | | |
| Referral nudges  *Targeting HCPs* |  | Does your patient have a confirmed diagnosis of a chronic lung disease?   - Are they limited by breathlessness on physical activity? - Are their medications optimised? - Are they safe to exercise?   If you answered yes to any of the above,  refer this patient to Pulmonary Rehabilitation by completing an eReferral:  **Physiotherapy > Pulmonary Rehabilitation**  How you ‘sell’ the program to patients is important – **be enthusiastic** when speaking about Pulmonary Rehabilitation | |  |
|  | Referral nudges were placed on each computer in respiratory medicine clinics and on the respiratory ward. This information was also included in the rotational respiratory medicine orientation packs and was re-iterated by respiratory physiotherapists. | | | |
| Physiotherapy resources  *Targeting people with COPD* | Lung Foundation Australia fact sheet (available here: [Pulmonary rehabilitation - Lung Foundation Australia](https://lungfoundation.com.au/support-resources/resource-hub/pulmonary-rehabilitation/#:~:text=The%20Pulmonary%20Rehabilitation%20fact%20sheet%20provides%20an%20overview,safe%3F%20What%20topics%20are%20in%20the%20education%20sessions%3F)), and WA health *Living better with exercise* booklet | | | |
| PRP education to rotating staff  *Targeting HCPs* | Education delivered by PRP physiotherapists to medical staff, junior/newly rotated ward physiotherapists, respiratory nurses. Referrers to attend an assessment and a PRP class. | | | |
| Changes to referral processes  *Targeting people with COPD* | Once patients referred to the program:   - Mail out PRP resources (invitation letter, program brochure, infographic) to patients within two weeks of referral - Utilise ‘did not respond’ letter for those who do not respond - Physiotherapist makes phone contact with patient prior to initial assessment | | | |

Footnotes: Downloadable copies of co-designed strategies are available on the Open Science Framework project page for this study.^8^ eReferral is the electronic referral system used to generate referrals to all services within Western Australia Health. *This was decided to encourage behaviour change in the treating HCP rather than to give the role directly to the case finder who would likely discontinue beyond the study completion due to funding restrictions.

Abbreviations:

COPD – chronic obstructive pulmonary disease

HCP – healthcare professional

PRP – Pulmonary rehabilitation program

**References**

1.	Leask CF, Sandlund M, Skelton DA, et al. Framework, principles and recommendations for utilising participatory methodologies in the co-creation and evaluation of public health interventions. *Res Involv Engagem* 2019; 5: 2. 2019/01/18. DOI: 10.1186/s40900-018-0136-9.

2.	Milner SC, Boruff JT, Beaurepaire C, et al. Rate of, and barriers and enablers to, pulmonary rehabilitation referral in COPD: a systematic scoping review. *Respir Med* 2018; 137: 103-114. DOI: 10.1016/j.rmed.2018.02.021.

3.	Watson J, Jordan R, Gardiner L, et al. A systematic review of the effectiveness of interventions to promote referral; adherence; and uptake of pulmonary rehabilitation for patients with chronic obstructive pulmonary disease. *Int J Chron Obstruct Pulmon Dis* 2023; 18: 1637-1654. DOI: 10.2147/COPD.S396317.

4.	Hug S, Cavalheri V, Gucciardi DF and Hill K. An evaluation of factors that influence referral to pulmonary rehabilitation programs among people with COPD. *CHEST* 2022; 162: 82-91. DOI: 10.1016/j.chest.2022.01.006.

5.	Hug S, Cavalheri V, Hill K and Gucciardi DF. Road to referral success in COPD: enhancing patient engagement through dedicated conversations about pulmonary rehabilitation programs. *Respir Med* 2024; 233: 107790. 20240830. DOI: 10.1016/j.rmed.2024.107790.

6.	Mental Health Commission. *Working together toolkit: designed to support the practical implementation of the Mental Health and Alcohol and Other Drug Engagement Framework 2018–2025*. 2018. Perth: Government of Western Australia.

7.	International Collaboration for Participatory Health Research (ICPHR). *Position paper 1: what is participatory health research?* May 2013. Berlin: ICPHR.

8.	Hug S, Hill K, Gucciardi D and Cavalheri V. Interrupted time series study, https://osf.io/mzn4v/?view_only=4ec1376816ba462d9f04bd53dd7dd4b9 (2024, accessed 5 March 2024).

9.	Hill K, Cavalheri V, Gucciardi DF and Hug S. How can healthcare professionals promote pulmonary rehabilitation in people with COPD? A qualitative study. *Patient Educ Couns* 2025; 136: 108781. 20250408. DOI: 10.1016/j.pec.2025.108781.

10.	Deci EL, Eghrari H, Patrick BC and Leone DR. Facilitating internalization: the self-determination theory perspective. *J Pers* 1994; 62: 119-142. DOI: 10.1111/j.1467-6494.1994.tb00797.x.

11.	Ryan R, Patrick H, Deci E and Williams G. Facilitating health behavior change and its maintenance: interventions based on self-determination theory. *Eur Health Psychol* 2007; 10: 2-5.

12.	Teixeira PJ, Marques MM, Silva MN, et al. A classification of motivation and behavior change techniques used in self-determination theory-based interventions in health contexts. *Motiv Sci* 2020; 6: 438-455. DOI: 10.1037/mot0000172.

13.	Hug S, Cavalheri V, Gucciardi DF and Hill K. Resources for research assistants, https://osf.io/qet25/?view_only=e185130575a54e2aa4d95435977dda1a (2024, accessed 5 March 2024).
