# Supplementary material for: Implementation of co-designed strategies enhances referral to pulmonary rehabilitation for people with COPD in tertiary care: an interrupted time series study
Source: Implement Sci. 2026 Jun 10;21:46. doi: 10.1186/s13012-026-01512-4 (PMC13483743; doi:10.1186/s13012-026-01512-4)
Supplement: Supplementary file 2 [file 13012_2026_1512_MOESM2_ESM.docx]

**Supplement 2 – Net cost impact**

(A) Annual cost of co-designed strategies

To explore the annual cost of the co-designed strategies, we recorded costs associated with deploying each of the co-designed strategies during the 5-month post-implementation period across the three tertiary hospitals. The commencement of the post-implementation period was initially planned for January 2022; however, this was delayed until March 2023 due to hospital lockdowns imposed by the COVID-19 pandemic. Because of this delay certain strategies were implemented twice, and it is likely that the total cost was over-estimated.

During the post-implementation period, case finders were deployed at each hospital. Each case finder completed weekly timesheets documenting hours worked onsite. At the end of the period, we calculated the average full-time equivalent (FTE) for a case finder at each site to estimate employment costs. This FTE accounted for salary and included the standard 24% on-costs stipulated by the Western Australian Department of Health. Across all sites, the combined FTE was 1.28—comprising 0.37 FTE at Site A, 0.56 FTE at Site B, and 0.35 FTE at Site C. The case finder salary was costed at a typical entry-level physiotherapist (P1.2), which was AUD$85,169 as of 1 July 2023. ^1^ After adjusting for the 1.28 FTE, this equated to an annual salary of AUD$109,016. With the 24% on-costs applied, the total was AUD$129,457.88.

The total cost of delivering these co-designed strategies was AUD$147,975. A breakdown of the costs is provided below:

| Item | Itemised cost (AUD) | Total cost per annum (AUD) | |
| --- | --- | --- | --- |
| Co-designed strategies | Printing (LFA factsheets): $685  Printing (Infographics): $737  Clinic posters: $290  Clinician polo shirts: $1675  Parking vouchers: $5940  Printing (living better with lung disease books): $5100  Lung’s logo: $200  Lung foundation fans: $3890  Case finders: $129,458 | $147,975 |  |

(B) Cost of delivering a PRP (per person, per class)

We then estimated the cost of delivering a pulmonary rehabilitation program (PRP) for those who were successfully referred to the service. Staffing costs were based on a senior physiotherapist (P2.3), assuming four hours per week to run two PRP classes, with an additional 25% time allocated for assessments and administrative support, plus four hours annually for mandatory training. In addition to salary costs, we included equipment expenses related to PRP delivery. These comprised one-off setup costs and annual maintenance for items such as the Massimo device, stethoscope, blood pressure monitor, stopwatches, free weights, clipboards, and trolleys.

The cost of delivering a PRP was estimated at AUD$21,880 in the first year, and AUD$20,092.08 per annum for subsequent years where equipment could be reused. Given that participants were referred to already established PRPs, the staffing, administration, training, and ongoing equipment cost were used in the analysis. A breakdown of the costs is provided below:

| Item | Itemised cost (AUD) | Total cost per annum (AUD) |
| --- | --- | --- |
| PRP delivery | Staffing: $15,594  Administration time: $3,898  Mandatory training time: $300  Equipment: $2,088 (initial)  Equipment: $300 (ongoing) | Initial $21,880  Ongoing $20,092 |

In terms of class costs, the capacity was for 10 patients per class, which resulted in a cost of AUD$1,005 per class ($20,092 / 20). With an average attendance of 7 patients per class, the cost per class rose to AUD$1,435 ($20,092 / 14). If the class runs twice a week for 50 weeks per year, the cost per client per class would be AUD$20.09 for full attendance (10 patients per class) and AUD$28.70 for 70% attendance (7 patients per class).

(C) Cost per additional person referred

We estimated that there would be 295 broadly eligible people with COPD who present to tertiary care across a 5-month period (average number recruited over the pre- and post-implementation periods – i.e. 391+199/2). This equates to 59 people referred per month (295/5), and 708 eligible people referred per year (59x12). During the pre-implementation period, the usual referral rate was 25%, meaning that 25% of the eligible population was typically referred to the program. Applying this referral rate to the estimated total number of eligible people per year yields 177 referrals per annum during the pre-implementation period (708 × 0.25). After the implementation of co-designed strategies, the referral rate increased to 52%, resulting in 368 referrals per annum during the post-implementation period (708 × 0.52). The increase in referrals with these strategies in place was estimated at 191 per annum (368 post-implementation referrals minus 177 pre-implementation referrals).

Each person costs between AUD$20.09 to AUD$28.70 per class (as calculated above for full or 70% attendance). Considering this cost and the additional 191 people with COPD referred to a PRP with the co-designed strategies in place, the estimated total cost for delivering a PRP for these additional people ranges between AUD$3,837 (191 x AUD$20.09) and AUD$5,485 (191 x AUD$28.70) per annum, based on attendance.

Considering the cost of delivering the co-designed strategies (A) and the cost per additional person referred (C), the health department would need to allocate a total of AUD$151,812 to AUD$ $153,460 per annum, to cover both costs.

(D) Cost per hospitalisation

The costs of hospitalisation in people with COPD can be significant as demonstrated by the Australian national hospital cost weights for frequently used items. ^2^ For example, 2021 data demonstrate that E65A (Respiratory infections and inflammations, major complexity) has an estimated cost of per patient, per admission of AUD$9,410 and E65B (Respiratory infections and inflammations, minor complexity) has an estimated cost of per patient, per admission of AUD$4,432. ^2^ To express these values in 2025 Australian dollars, we applied the Australian Bureau of Statistics (ABS) health inflation rate. ^3^ Based on the latest ABS data, the annual inflation rate for the health category was 4.1% in the 12 months to March 2025. Assuming this rate remained constant from 2021 to 2025, the inflation factor over 4 years is approximately 1.174 (1.041^4^). The cost weights with this adjustment applied are estimated as AUD$11,050 and AUD$5200 for E65A and E65B, respectively (9410 × [1.041]^4^ and 4432 × [1.041]^4^).

Across Australia in 2017-18 there were 77,754 COPD-related hospitalisations, of which, 6,499 occurred in Western Australia. ^4, 5^ Pulmonary rehabilitation is understood to reduce hospitalisation risk. ^6^ To be conservative, we used the upper estimate of the total costs (AUD$153,460) and calculated that we would need to prevent 14 hospitalisations coded as a major complexity, or 30 hospitalisations coded as a minor complexity, per annum for the co-designed strategies and additional PRP delivery to be cost neutral.

**References**

1.	Commission WAIR. *WA Health System - HSUWA – Pacts Industrial Agreement 2022*. 2022.

2.	Authority IHaACP. *National Hospital Cost Data Collection*. 2021.

3.	(ABS) ABoS. Consumer Price Index, Australia, https://www.abs.gov.au/statistics/economy/price-indexes-and-inflation/consumer-price-index-australia (2025, accessed 7 July 2025).

4.	Australian Institute of Health and Welfare (AIHW). *Chronic obstructive pulmonary disease*. 2023. Canberra: AIHW.

5.	Australian Commission on Safety and Quality in Health Care (ACSQHC). *Fourth Atlas 2021 - 2.1 Chronic obstructive pulmonary disease (COPD)*. 2021.

6.	Griffiths TL, Burr ML, Campbell IA, et al. Results at 1 year of outpatient multidisciplinary pulmonary rehabilitation: a randomised controlled trial. *Lancet* 2000; 355: 362-368. 2000/02/09. DOI: 10.1016/s0140-6736(99)07042-7.
